# Supplementary material for: Hierarchically Porous Carbon Networks Derived from Chitosan for High-Performance Electrochemical Double-Layer Capacitors
Source: Nanomaterials (Basel). 2023 Nov 16;13(22):2961. doi: 10.3390/nano13222961 (PMC10675306; doi:10.3390/nano13222961)
Supplement: Supplementary file 1 [file nanomaterials-13-02961-s001.zip › nanomaterials-2705183-supplementary.pdf]

Supporting Information for

Hierarchically porous carbon networks derived from chitosan for high-performance electrochemical double-layer capacitor

Kwang Hyun Park[1,†], Segi Byun[2,3,†], Boemjin Ko[1], Woong-Gil Hong[4], Jungmo Kim[5], Dongju Lee[6,*], Wang Geun Shim[4,*], and Sung Ho Song[1,*],

[1]Division of Advanced Materials Engineering, Center for Advanced Materials and Parts of Powders, Kongju National University, Cheonan-si 31080, Republic of Korea

[2]Hydrogen Convergence Materials Laboratory, Korea Institute of Energy Research (KIER), 152 Gajeong-ro, Yuseong-gu, Daejeon, 34129, Republic of Korea

[3]Energy Engineering, University of Science and Technology (UST), Daejeon, 34113, Republic of Korea

[4]Department of Chemical Engineering, Sunchon National University, 255 Jungang-Ro,

Suncheon-si 57922, Republic of Korea.

[5]Nano Hybrid Technology Research Center, Korea Electrotechnology Research Institute (KERI), Changwon, 51543, Republic of Korea

[6]Department of Urban, Energy, and Environmental Engineering, Chungbuk National University, Chungdae-ro 1, Seowon-Gu, Cheongju, Chungbuk 28644, Republic of Korea

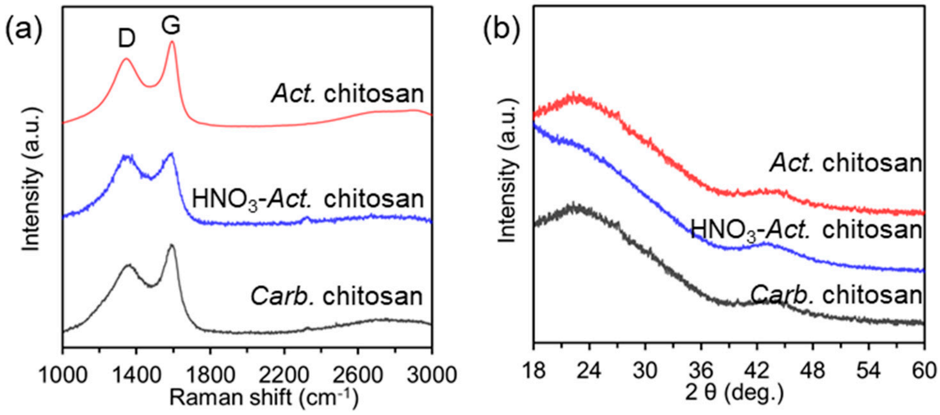

Figure S1. Raman spectroscopy and X-ray diffraction patterns of Carb. Chitosan, HNO_3-*Act.* Chitosan. (a) Raman spectroscopy. (b) XRD patterns.

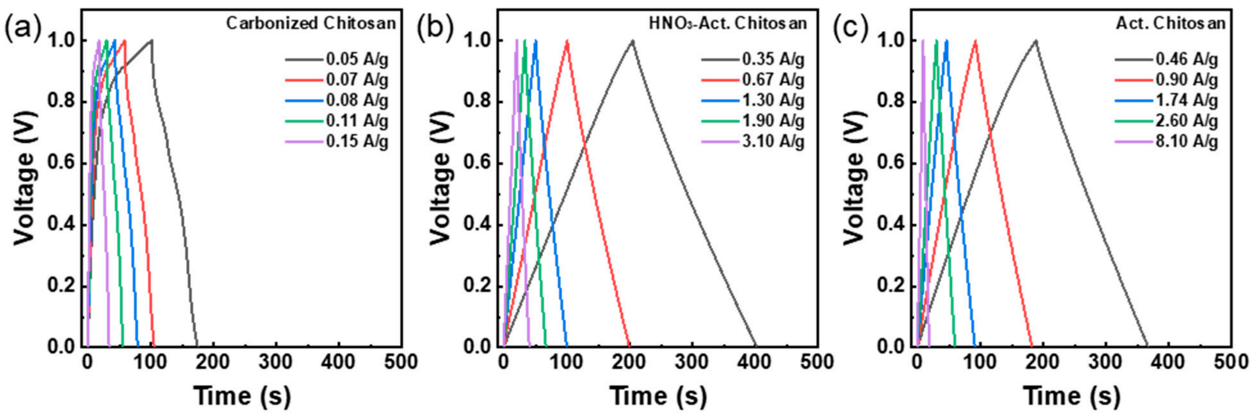

Figure S2. Galvanostatic charge/discharge (GCD) curves of (a) *Carb.* Chitosan, (b) HNO_3-*Act.* Chitosan and (c) *Act.* Chitosan samples at a function of current densities.

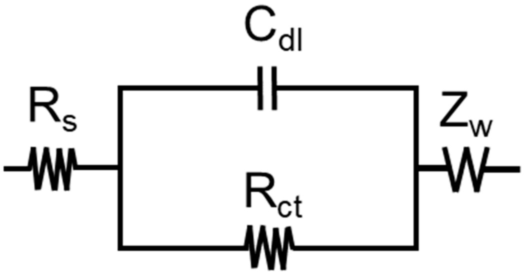

Figure S3. Equivalent circuit model for fitting of the Nyquist plot for various carbon samples. R_s: series resistance, R_{ct}: charge transfer resistance, C_{dl}: double layer capacitance, and Z_w: Warburg impedance.

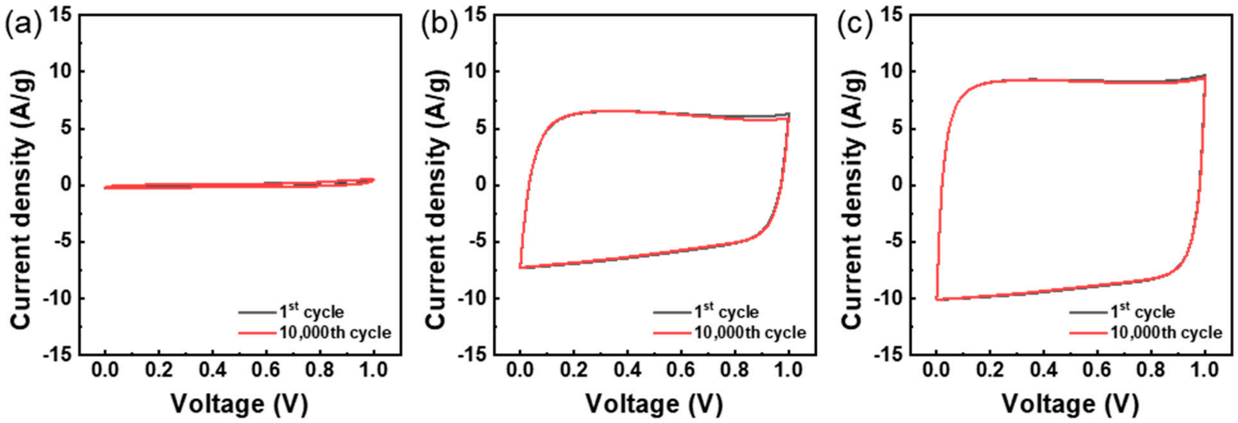

Figure S4. CV curves collected at the first and after 10,000 cycles. (a) *Carb.* Chitosan, (b) HNO_3-*Act.* Chitosan and (c) *Act.* Chitosan

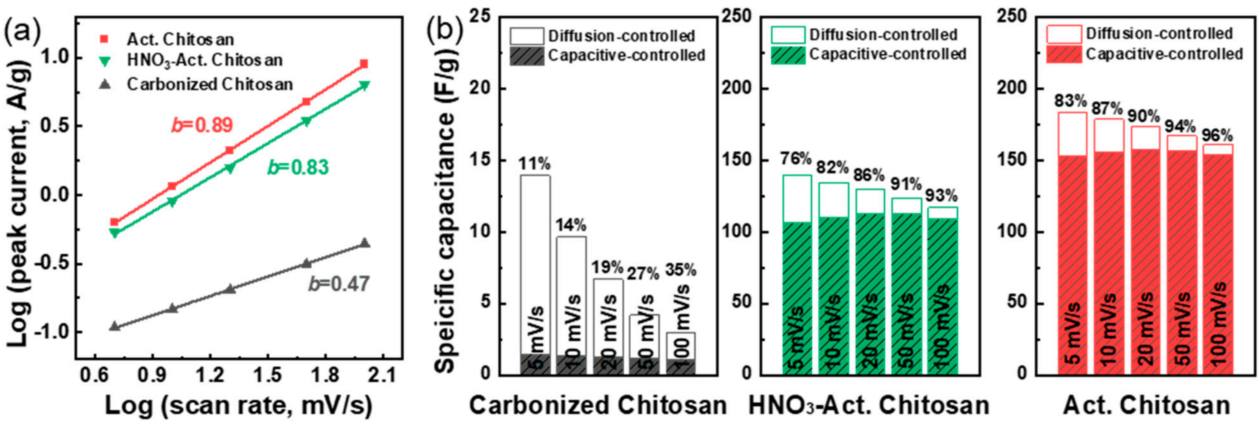

Figure S5. Charge storage mechanisms. (a) log(I) vs. log(v) plots of anodic current at 1.0 V. (b) Capacitance comparisons of *Carb.* Chitosan, HNO_3-*Act.* Chitosan, and *Act.* Chitosan samples with capacitive contribution at a scan rate from 5 to 100 mV/s.

Table S1. Detailed Nyquist plot fitting results for various carbon samples.

Sample	R_s (Ω)	R_{ct} (Ω)
Carb. Chitosan	2.694	458.2
HNO_3-*Act.* Chitosan	3.667	0.438
Act. Chitosan	2.807	0.395
